# Oxygen Transfer Effect on the Growth of *Limosilactobacillus reuteri* ATCC 53608 and on Its Metabolic Capacity

**DOI:** 10.1007/s00284-024-03822-6

**Published:** 2024-09-17

**Authors:** Sandra-Janneth Santos-Rocha, Cristian Mendoza-Ortiz, Julian Tobon-Gonzalez, Rigoberto Ríos-Estepa, Fernando Orozco-Sánchez

**Affiliations:** Facultad de Ciencias, Universidad Nacional de Colombia, Medellín, Colombia

## Abstract

*Limosilactobacillus reuteri* is a probiotic microorganism used in the treatment of gastrointestinal disorders. The effect of oxygen transfer on cultures of *L. reuteri* ATCC 53608 at shake flask and stirred tank bioreactor scales was studied, using MRS and molasses-based media. At shake flask scale, in MRS medium, a maximum bacterial concentration of 2.01 ± 0.02 g L^−1^ was obtained; the oxygen transfer coefficient was 2.01 ± 0.04 h^−1^. Similarly, in a 7.5 L bioreactor, in MRS, a maximum bacterial concentration of 2.46 ± 0.16 g L^−1^ was achieved (k_L_a = 2.64 ± 0.06 h^−1^). In contrast, using a molasses-based medium, bacterial concentration reached 3.13 ± 0.17 g L^−1^ in the 7.5 L bioreactor. A progressive reduction in lactic acid concentration and yield was observed as the oxygen transfer coefficient increased, at shake flask scale. Also, the oxygen transfer coefficient strongly affected the growth of *L. reuteri* in shake flask and bioreactor and allowed us to successfully scale up *L. reuteri* culture, producing similar maximum bacterial concentrations in both scales (2.01 g L^−1^ and 2.46 g L^−1^ in MRS). This is the first study on oxygen transfer coefficients in *L. reuteri,* and it is a valuable contribution to the field as it provides important insights about how this organism tolerates oxygen and adapts its metabolism for larger biomass production.

**Graphical Abstract:**

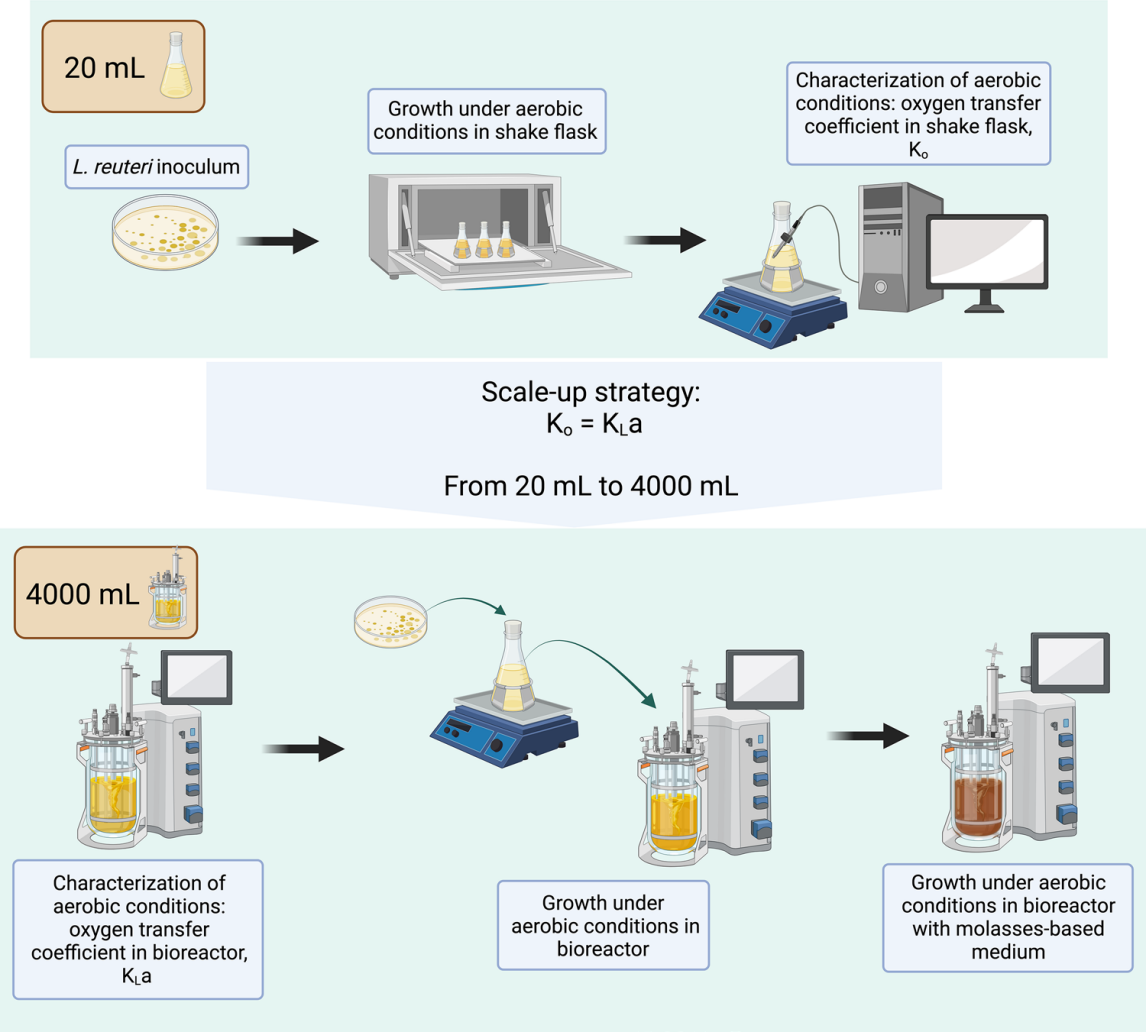

## Introduction

Lactic acid bacteria (LAB) have been widely used in research and industry. It constitutes a diverse group of microorganisms commonly associated with plants, meat, and dairy products. Several species are well known due to their probiotic properties [1]. *Limosilactobacillus reuteri* (*L. reuteri*) is a Generally Recognized as Safe (GRAS) microorganism, which plays a crucial role in human health performing important functions throughout life [2]. In terms of digestion and nutrition, *L. reuteri* aids babies in digesting breast milk and solid foods, and strengthens the gut lining to prevent leakage and improve immune responses. It releases molecules that foster a healthy and diverse microbiota while inhibiting pathogen growth. For the immune system, *L. reuteri* benefits the host by reducing the production of pro-inflammatory cytokines, promoting regulatory T cell development and function, and fortifying the intestinal barrier to decrease microbial translocation and inflammation. Its antimicrobial activity includes producing organic acids, ethanol, and reuterin, which inhibits pathogenic microbes’ growth and remodels the host’s microbiota composition [3–5]. Moreover, *L. reuteri* exhibits antiviral and antifungal properties, effectively inhibiting species of *Candida* and combating various viruses such as pneumoviruses, circoviruses, rotaviruses, coxsackieviruses, and papillomaviruses. Evolutionarily significant, *L. reuteri* is one of the few bacterial species that has co-evolved with humans, with specialized strains passed down through generations, enhancing human health across lifespans. Proven safe and effective for all ages, including infants and immunocompromised adults, even at high doses, *L. reuteri's* efficacy is well documented in diverse populations [6].

*L. reuteri* is a facultative anaerobe that, despite having an incomplete electron transport chain, can utilize oxygen as a final electron acceptor when heme and menaquinone are present, leading to increased biomass concentrations [7]. To fully benefit from the features of *L. reuteri*, bioprocess methodologies to enhance its biomass and metabolite production should be explored and researched. Aerobic conditions can enhance the growth, stress tolerance, and metabolic activity of LAB by allowing them to utilize oxygen for respiration, which increases their tolerance to heat and oxidative stress [8]. However, these conditions can also increase the production of reactive oxygen species (ROS) that damage cellular components. In this way, oxygen promotes reactive oxygen species (ROS) production [9, 10] leading to oxidative stress and growth limitations, therefore, the bioprocess must control oxygen concentration. To counteract this stress, lactic acid bacteria possess enzymes like catalase and superoxide dismutase to remove ROS [7, 11, 12]. Lab experiments in stirred tank bioreactors with *L. reuteri* ATCC 23272 showed a lower growth rate but larger biomass production at low oxygen concentrations compared to anaerobic conditions [13]. Despite these findings, aerobic bioprocess in lactic acid bacteria has been poorly studied and characterized. To our knowledge, oxygen mass transfer coefficients (k_L_a) have not been utilized to characterize and scale up biomass production of *L. reuteri*. The study of these coefficients as a scaling-up criterion has facilitated the establishment of industrial biotechnological processes and the production of high-value metabolites [8, 14, 15]. The goal of this study was to characterize and evaluate the effect of oxygen transfer on aerobic cultures of *L. reuteri* ATCC 53608 at shake flask and bioreactor scales, using different culture media. The study also aimed at assessing if *k*_L_*a* could be used as a scaling-up criterion for this biological system. Further, the potential use of molasses-based culture media for industrial biomass production of *L. reuteri* ATCC 53608 was also considered.

## Materials and Methods

### Microorganism

The lyophilized *L. reuteri* strain ATCC 53608 was obtained from the American Type Culture Collection (ATCC) and reactivated according to the ATCC recommendations. In order to generate anaerobic atmosphere, Oxoid™ AnaeroJar™ 2.5 L Thermo Scientific™ was used with the gas generating sachets (Oxoid™ AnaeroGen™ sachet). Colonies were obtained from this bacterial suspension growing in MRS broth in MRS broth—Merck 1.10661 [16], incubated at 37 °C under anaerobic conditions for 48 h. Then, colonies were stored in vials under freezing conditions at −70 °C in a 20% glycerol solution. We periodically conducted contamination tests and estimated the bacterial concentration in these suspensions to ensure contaminants-free vials, and bacterial concentrations greater than 10^8^ CFU mL^−1^. A vial was periodically reactivated in solid MRS medium for 48 h and stored at 8 °C as working material.

### Cultivation of *L. reuteri* in MRS Broth at Shake Flask Scale

Cultures were carried out in 100 mL flasks containing 20 mL of MRS broth using cotton closures. Flasks were inoculated at a final concentration equivalent to 0.1 optical density (OD) at 600 nm. The suspensions were incubated at 37 °C and at 0, 80, 120, 200, and 250 revolutions per minute (rpm). For sampling purposes, 3 flasks were removed every 2 h, to measure biomass production, substrate consumption, and product formation.

### Estimation of the Oxygen Transfer Coefficient at Shake Flask Scale (*Ko*)

The oxygen transfer rate (OTR) in shake flasks can be represented by Eq. 1 [17, 18]:

1 $$OTR = K_{0{ }} \left( {C_{O2,air} - C_{O2} } \right),$$

where C_O2, air_ (kg m^−3^) is the oxygen concentration in the liquid phase at equilibrium with the air at the temperature of the system, C_O2_ (kg m^−3^) is the oxygen concentration in liquid phase, and *Ko* (h^−1^) is the overall mass transfer coefficient for a shake flask (including the transfer through the plug and the liquid–gas interface). Considering the previous equation, the dynamic method (Eq. 2) for the estimation of *Ko* in the different culture conditions and without cells was used [17], [19]

2 $${K}_{0}\left(t-{t}_{i}\right)=ln\left(\frac{{C}_{O2,ss}-{C}_{O2,i}}{{C}_{O2,ss}-{C}_{O2}}\right),$$

where C_O2,ss_ is the steady-state oxygen concentration in the liquid medium without cells, C_O2,i_ is the oxygen concentration at the initial time t_i_, and C_O2_ is the oxygen concentration at the time t. Measurements were carried out in a system consisting of a dissolved oxygen sensor (Applisens Model Z1023525). The system was adjusted at the corresponding agitation speed, and then, oxygen was displaced both from the liquid and from the headspace, using nitrogen, until DO values were lower than 10% saturation. Once the concentration of dissolved oxygen started to increase in the culture medium, values between 20 and 60% of DO were recorded. The *Ko* value was determined by linear regression, taking (t–t_i_) as the abscissa axis and the term of the natural logarithm as the ordinate axis. This method allows to determine the global oxygen transfer coefficient (Ko) or the offered oxygen—oxygen transfer rate (OTR)—by the shake flask to the cells, considering two mass transfer resistances: the resistance in the plug and the resistance in the liquid–gas interface inside the flask [17]. Ko is comparable to k_L_a for the scaling up process (from shake flask to bioreactor), as it will be later explained.

### Cultivation of *L. reuteri* in MRS Broth at Bioreactor Scale

Experiments were carried out in a New Brunswick 7.5 L stirred tank bioreactor with a working volume of 4 L, equipped with a pH probe, an oxygen probe, a temperature sensor, a Rushton turbine, a 7-hole diffuser, and baffles. The temperature and pH were controlled at 37 °C and 5.5, respectively. The latter was controlled with the addition of either 3 M KOH or 3 M H_3_PO_4_, as required. A culture of *L. reuteri* ATCC53608 in MRS broth incubated for 16 h at 120 rpm and 37 °C was used as inoculum. The bioreactor was inoculated with a biomass concentration equivalent to 0.1 optical density at 600 nm. Samples were taken every 2 h for 16 h to determine biomass, glucose, and lactic acid concentrations. The stirring speed and aeration flow rate were adjusted until achieving a similar oxygen transfer coefficient to that obtained in the previous tests, using shake flask. Thus, three operation conditions were studied with k_L_a values of 2.64, 5.22, and 14.04 h^−1^. For this, operating conditions were set at 0.05 vvm, and an agitation rate between 225 and 312 rpm; the minimum k_L_a value (with aeration), obtained in the stirred tank bioreactor was 2.64 h^−1^.

### Determination of the Oxygen Transfer Coefficient at Bioreactor Scale (k_L_a)

The OTR in stirred tank bioreactors can be represented by Eq. 3 [19])

3 $$OTR={k}_{L}a\left({C}_{O2,air}-{C}_{O2}\right),$$

where ${C}_{O2,air}$ and ${C}_{O2}$ were previously defined. k_L_a, (h^−1^), is the mass transfer coefficient from the air bubbles to the culture medium. Considering Eq. 3, the dynamic method was used to determine *k*_L_*a* values [19]. The air flow rate and the agitation rate were adjusted to achieve the oxygen transfer coefficients previously described (2.64, 5.22, and 14.04 h^−1^). It is important to highlight that k_L_a was determined prior to any cultivation, bearing in mind any susceptible effect of changes in the composition of the medium, and, in the configuration of the bioreactor, during sterilization.

### Cultivation of *L. reuteri* in a Molasses-Based Medium

A pretreatment step for the sugar cane molasses was necessary to remove suspended solids that could promote contamination. A mixture of 50% molasses and 50% MQ water was boiled, allowed to cool, and then left to precipitate for 24 h. The clarified liquid was carefully separated from the precipitated solids and used as a stock solution for culture media preparation. Table 1 shows the composition of the molasses-based culture medium used during the various experiments. This medium was supplemented with the same salts and nitrogen content as the MRS medium. Bioreactor assays with the molasses-based medium were carried out under the same cultivation conditions as those for the MRS broth.

**Table 1:** Composition of the economical medium used for cultivation of *L. reuteri*

Component	Concentration (g L^−1^)
Sucrose (C_12_H_22_O_11_)^a^	20.00
Yeast extract^b^	20.50
Dipotassium hydrogen phosphate^b^ (K_2_HPO_4_)	2.00
Tween® 80^b^	1.00
Triammonium citrate^b^ (C_6_H_17_N_3_O_7_)	2.00
Sodium acetate trihydrate^b^ (C_2_H_3_NaO_2_*3H_2_O)	5.00
Magnesium sulfate heptahydrate^b^ (MgSO_4_*7H_2_O)	0.20
Manganese sulfate tetrahydrate^b^ (MnSO_4_*4H_2_O)	0.05

^a^Component from sugar cane molasses. The molasses was clarified according with the showed methodology and corresponds to 38.2 g concentrate molasses/L economic medium

^b^Added components

Bioreactor assays with the molasses-based medium were carried out under the same cultivation conditions as those for the MRS broth.

### Analytical Methods

Glucose concentration was determined by means of a TRU-life glucometer (OK Biotech Co., Ltd.). A drop of medium (or its dilution, if necessary) is placed on the test strip. This test strip works in a glucose range of 0.2 to 6 g/L. The test trip is placed on the monitoring system for quantitative measurement during 30 s. Sucrose and lactic acid concentrations were quantified through the RQflex plus reflectometer (Merck®). To determine biomass production, optical density measurements, using a spectrophotometer (Thermo Fisher Scientific 4001/4) were correlated with the dry biomass weight by means of a calibration curve: Dry Weight (g DW/L) = 0.4983 × optical density—0.0452 (R^2^ = 09946). This curve was prepared with a suspension of microorganisms in MRS broth. Optical density values of different dilutions of the culture were measured and the dry weight of the original suspension was determined through membrane filtration (MS® nylon filter, pore size: 0.22 μm, diameter: 47 mm) and dried at 105 °C for 2 h. For the case of the molasses culture, biomass production was determined by direct dry weight through membrane filtration, as previously described. Specific growth rates (μ_max_) were calculated using the 4-parameters logistic model, according to Eqs. 4 and 5 [20]:

4 $$X=a+ \frac{\text{b}}{1+{e}^{-\frac{t-c}{d}}}$$

5 $${\upmu }_{max}=\frac{\text{b}}{d{(\sqrt{a}+\sqrt{a+b})}^{2}},$$

where X represents the cell concentration (g L^−1^); t, cultivation time (h^−1^); a, b, c, and d, model parameters which were calculated using the Solver add-in of Microsoft Excel, adjusting the model to the experimental data. The doubling time (td) was calculated using Eq. (6). The maximum biomass (X_f_) also was calculated.

6 $${\text{t}}_{d}=\frac{\text{ln}(2)}{{\upmu }_{\text{max}}}$$

### Statistical Analysis

Experiments were performed in triplicate, each sample was analyzed three times. Mean values ± standard errors are reported. Statistically significant differences between groups were evaluated with one-way analysis of variance and Fisher’s least significant difference (LSD) procedure at a 95% confidence level.

## Results

### Cell Growth Kinetics and Oxygen Transfer in Cultivations with MRS Medium at Shake Flask Conditions

**Fig. 1:**
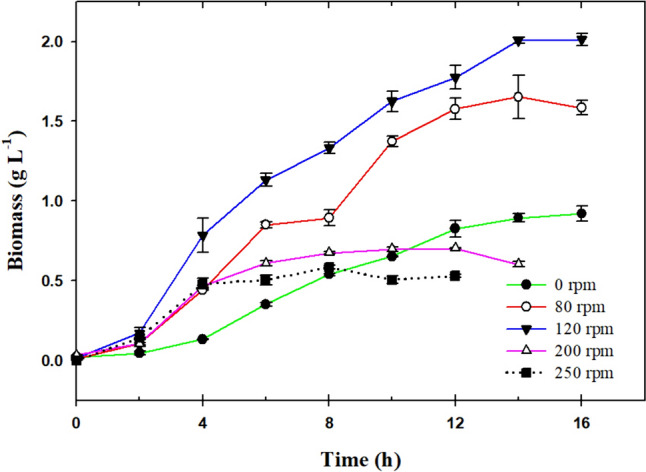
Dynamics of cell growth for *L. reuteri* ATCC 53608 in MRS broth at different agitation conditions in shake flask

Figure 1 depicts growth profiles for *L. reuteri* ATCC 53608 cultivations in shake flasks. As observed, the biomass production is highly dependent on the stirring condition. When agitation is increased, oxygen diffuses faster; turbulence is generated and mass transfer by convection greatly improves; mass transfer through plugs, headspace, and gas–liquid interface in flasks is also favored. For cell cultures at 0, 80, and 120 rpm (*Ko* measured in MRS = 0.60 ± 0.29 h^−1^, 1.77 ± 0,03 h^−1^, 2.01 ± 0,04 h^−1^, respectively), the stationary phase was reached at 14 h. Meanwhile, cultures at 200 and 250 rpm (*Ko* measured in MRS = 2.38 ± 0.06 h^−1^, 3.07 ± 0,07 h^−1^, respectively) reached the stationary phase earlier—at 6 h of cultivation—rendering faster and healthier cell growth; at this high oxygen concentration conditions, ROS might have been synthesized, which would affect cell metabolism, and even cause death and autolysis [21, 22]. Therefore, the largest biomass production of *L. reuteri* ATCC 53608 at shake flask scale in MRS broth (2.01 g L^−1^) was obtained at 120 rpm.

**Fig. 2:**
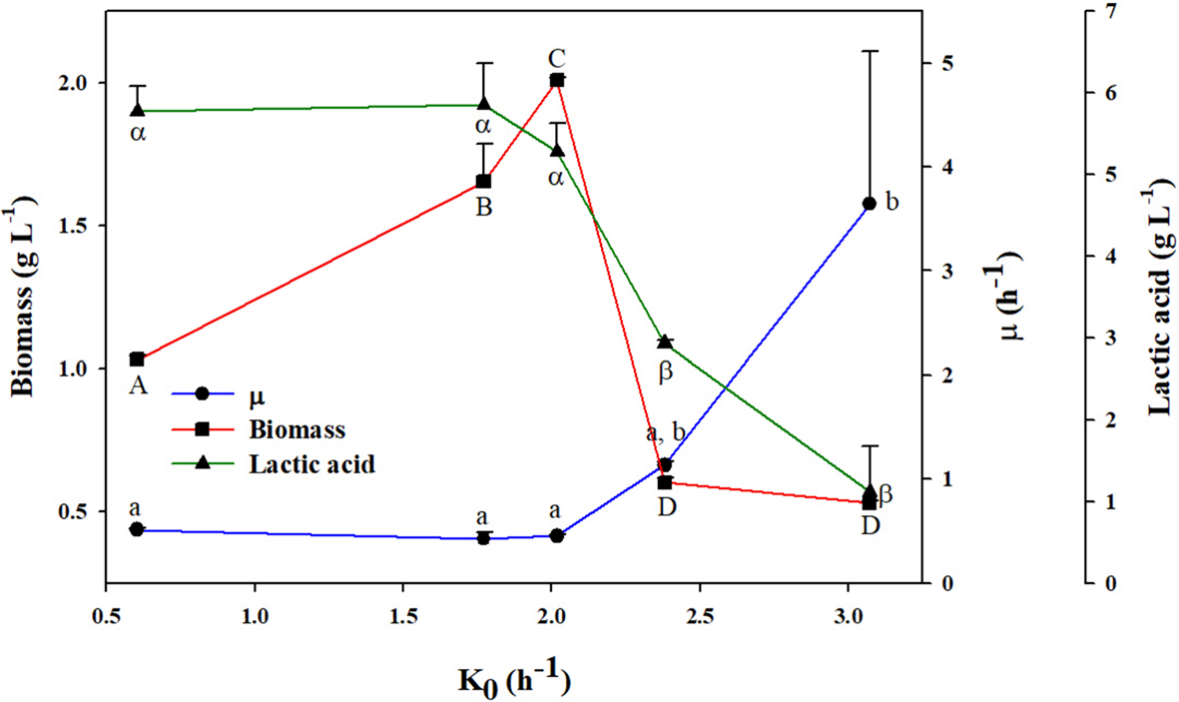
Effect of oxygen transfer on the growth of *L. reuteri* at shake flask scale. Data with the same letter are not statistically significant different, according to the Fisher’s least significant difference (LSD) procedure, at the 95.0% confidence level. Capital letter corresponds to biomass, lower case letter to specific growth rate to lactic acid

The lowest value of *Ko* obtained in this study was 0.60 ± 0.29 h^−1^, which corresponds to the absence of agitation (0 rpm); however, under this experimental condition, there is no such a strict anaerobic condition. As observed in Fig. 2, an increase in *Ko* produced an increase in biomass concentration until a maximum value of 2.01 g L^−1^ (which corresponds to a *Ko* value of 2.01 h^−1^). From this point on, the rise in the evaluated oxygen transfer coefficients triggered a tremendous reduction in cell growth (lower than 0.5 g L^−1^) at a *Ko* value of 2.38 h^−1^ (200 rpm) and 3.07 h^−1^ (250 rpm). Regarding the specific growth rates (μ_max_), these were maintained without significant differences for some values of the oxygen transfer coefficient (0.60 h^−1^, 1.77 h^−1^, and 2.01 h^−1^). Further, cultures with *Ko* of 2.38 h^−1^ and 3.07 h^−1^ rendered higher μ_max_ values, though biomass growth stopped very early compared to that of the other experimental conditions.

**Fig. 3:**
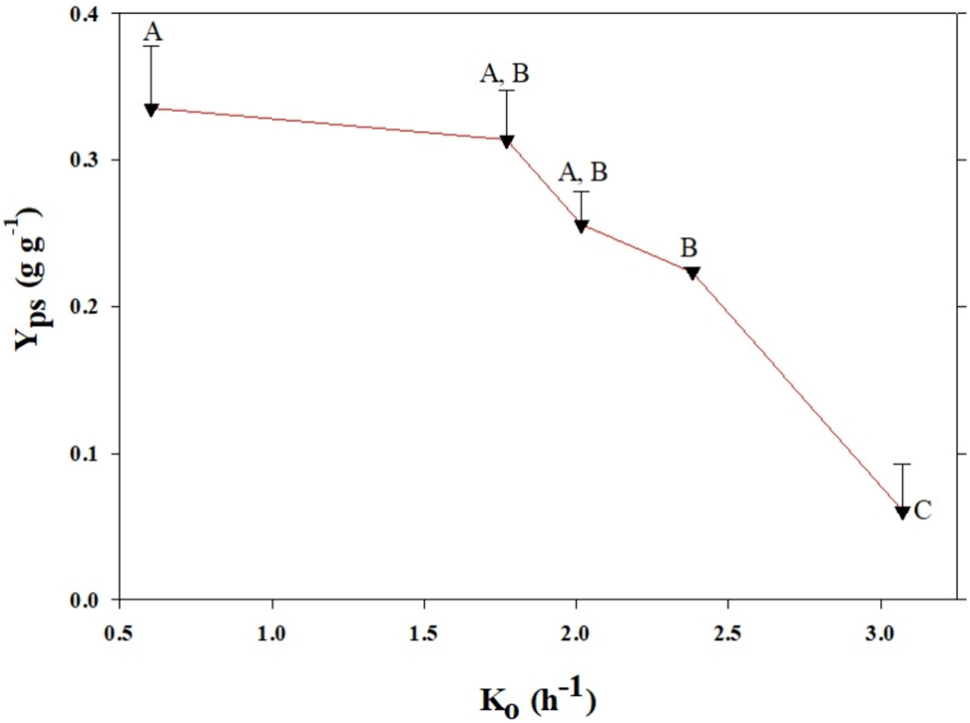
Effect of oxygen transfer on product yield (Y_ps_) of *L. reuteri* at shake flask scale. Data with the same letter are not statistically significant different, according to the Fisher’s least significant difference (LSD) procedure, at the 95.0% confidence level

Figure 3 shows the relationship between the oxygen transfer coefficient and product yield (lactic acid) Y_ps_. Biomass yield Y_xs_ was not influenced by oxygen concentration; values ranged from 0.046 ± 0.000 to 0.088 ± 0.042 g^−1^. There were no statistical differences in the mean value of Y_xs_; it remained statistically constant at different *Ko* values. Concerning the generation of lactic acid, the results showed that the higher the oxygen transfer, the lower the yield of this metabolite, Y_ps_. A maximum value of 5.28 ± 0.35 g L^−1^ of lactic acid was obtained at *Ko* value of 3.07 h^−1^.

### Change of Scale from Shake Flask to Bioreactor

**Fig. 4:**
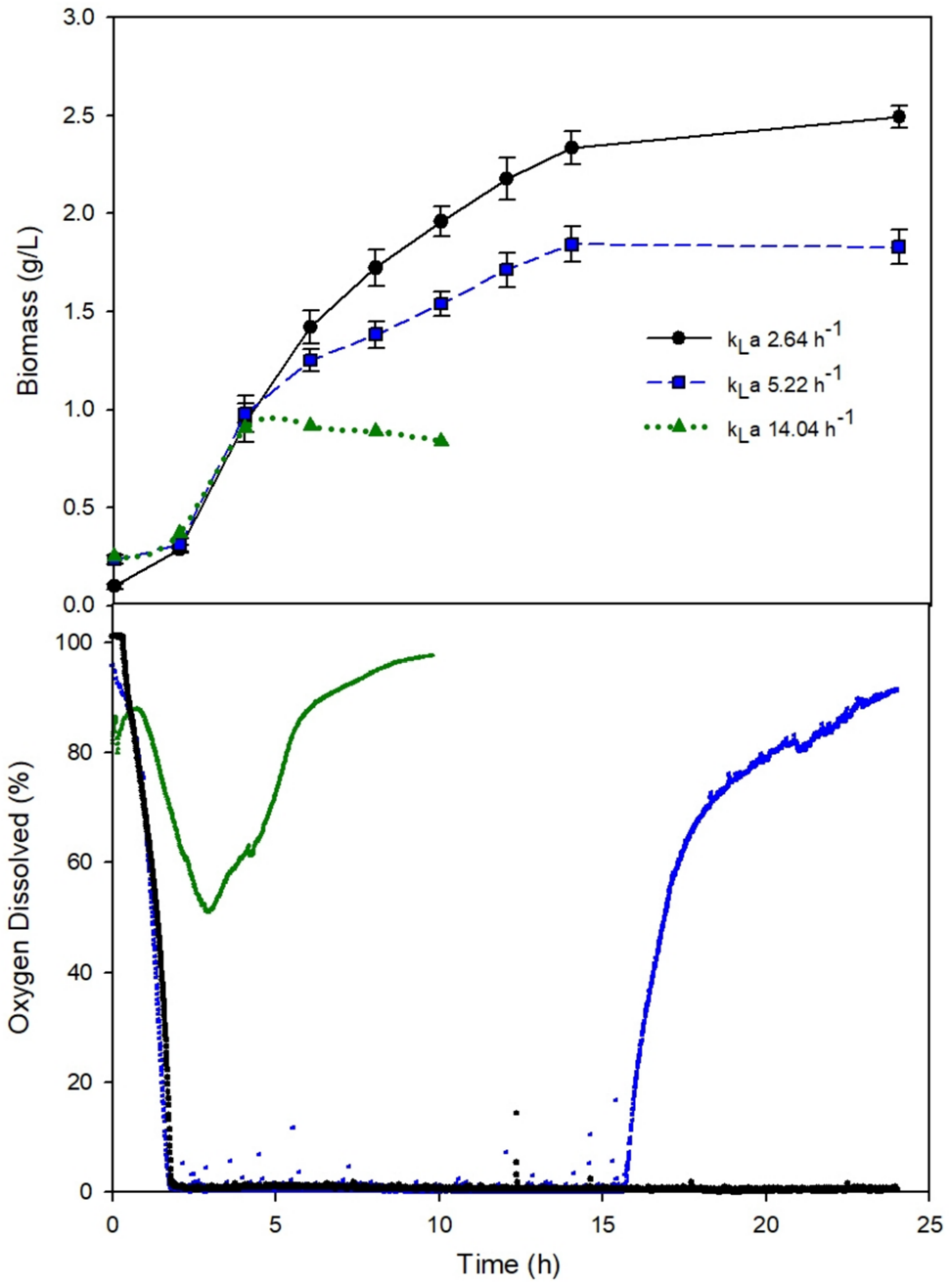
Dynamics of cell growth (A) and dissolved oxygen (B) for *L. reuteri* ATCC 53608 in MRS broth at different bioreactor k_L_a conditions

**Fig. 5:**
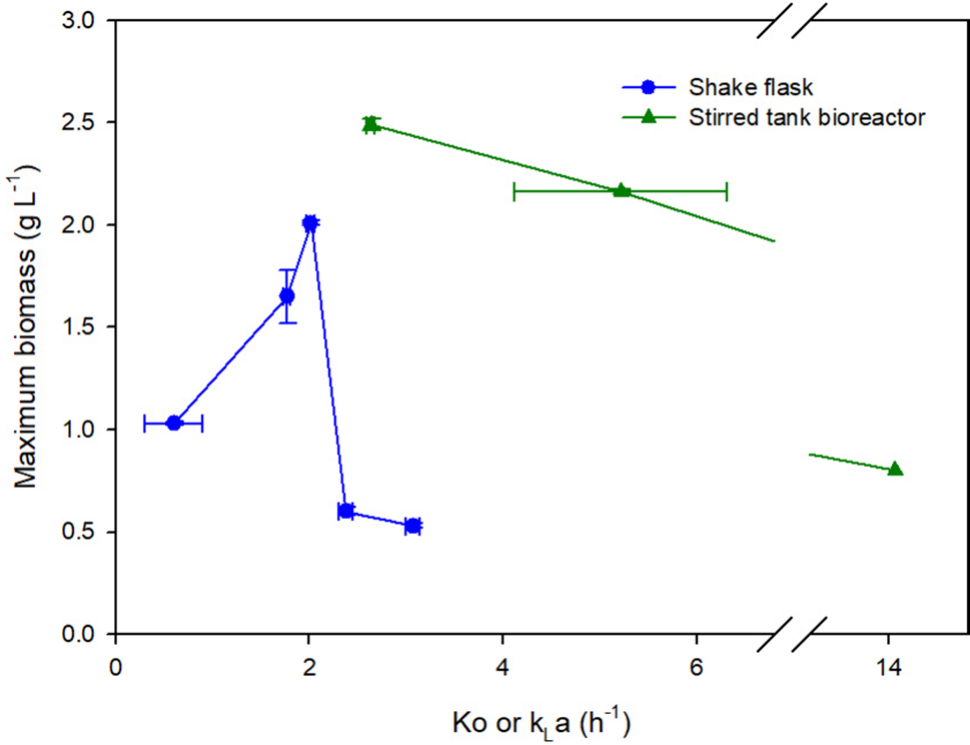
Effect of oxygen transfer on the growth of *L. reuteri* at shake flask (Ko) and stirred tank bioreactor (k_L_a)

The change of scale aimed at identifying the agitation and airflow conditions that would allow reaching a bioreactor k_L_a value similar to the *Ko* value obtained in flask cultures. The dynamics of cell growth and dissolved oxygen for *L. reuteri* ATCC 53608 in MRS broth at different bioreactor k_L_a conditions (2.64, 5.22, and 14.04 h^−1^) were evaluated, and are shown in Fig. 4. The relationship between the oxygen transfer coefficient and the growth of *L. reuteri,* at shake flask (Ko) and stirred tank bioreactor (k_L_a) conditions is presented in Fig. 5. Biomass production was higher in bioreactor than that in shake flask and followed a decreasing trend as k_L_a increased.

The closest k_L_a to *Ko* was 2.64 ± 0.06 h^−1^ obtained in MRS broths at 300 rpm and 0.05 vvm. Further, this k_L_a (2.64 h^−1^) resulted in the highest biomass production (2.46 g L^−1^). Interestingly, at this k_L_a, no accumulation of DO was observed, suggesting immediate consumption by biomass, which was maintained throughout most of the 16-h cultivation period. In contrast, cultures at k_L_a values of 5.22 h^−1^ and 14.04 h^−1^ produced only 0.8 g L^−1^ and 2.2 g L^−1^ biomass, respectively, and registered some DO accumulation and shorter cultivation periods, indicating a faster oxygen transfer rate than oxygen uptake rate by biomass.

**Fig. 6:**
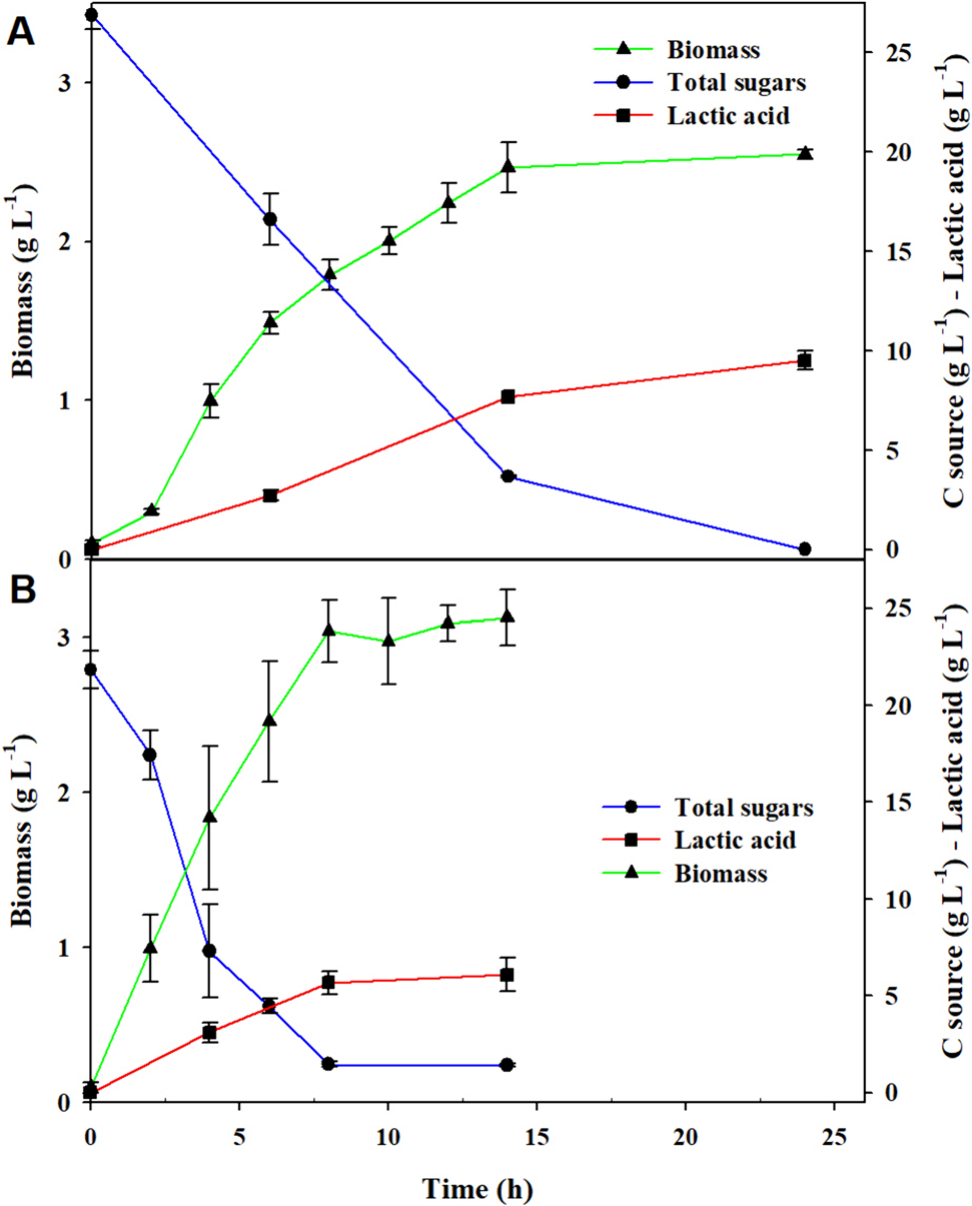
Dynamics of cell growth for *L. reuteri* ATCC 53608 at stirred tank bioreactor in **A** MRS broth and **B** an economical medium based on molasses

As it is observed in Fig. 6A, for cultures with MRS, the exponential phase ended at 14 h with a Y_ps_ of 0.398 ± 0.017 g g^−1^ and a biomass concentration of 2.46 ± 0.16 g L^−1^. Most of the kinetic parameters, originated from MRS at bioreactor scale, were similar to those values determined in shake flask, at conditions of maximum biomass production for each scale (Table 2), i.e., Ko = 2.01 ± 0.04 h^−1^ in shake flask and *k*_L_*a* = 2.64 ± 0.06 h^−1^ in bioreactor. Similarities also arise when comparing the specific growth rate (0.456 ± 0.016 h^−1^ and 0.481 ± 0.018 h^−1^, respectively). Regarding substrate consumption, a 75.94 ± 1.34% substrate assimilation was observed in flask, while 86.7 ± 0.3% was obtained in bioreactor. Moreover, the production of lactic acid in flask and in bioreactor were 5.28 g L^−1^ and 8.71 g L^−1^, respectively, which is consistent with the percentage of sugars assimilated. A higher consumption of glucose would render a larger product biosynthesis such as lactic acid (constant pH in bioreactor). Lactic acid concentration and Y_ps_ were (statistically) superior to those determined at flask scale, according to the Fisher’s least significant difference (LSD) procedure, at a 95.0% confidence level. Notwithstanding the differences in the declared parameters, the final biomass production from both experiments had no significant differences (*P* > 0.05) (values obtained were 2.01 ± 0.02 g L^−1^ in the flask and 2.46 ± 0.16 g L^−1^ in the bioreactor). Regarding biomass substrate yields Y_xs_, values were highly comparable (0.097 ± 0.005 g g^−1^ and 0.100 ± 0.008 g g^−1^ for flask and bioreactor, respectively). Yields of product from substrate (Y_ps_) were dissimilar, obtaining a value of 0.256 ± 0.023 g g^−1^ and 0.367 ± 0.021 g g^−1^ for flask and for bioreactor, respectively. Statistically significant differences (*P* < 0.05) might be caused by the biological and physical characteristics of each culture system, shake flask, and stirred tank bioreactor.

**Table 2:** Comparison between the kinetic parameters obtained for *L. reuteri* growing on MRS and molasses-based medium, at the maximum biomass production conditions

Parameter	MRS		Molasses medium
	Shake flask	Bioreactor	Bioreactor
Oxygen transfer coefficient (h^−1^)	Ko 2.01 ± 0.04	*k*_L_*a* 2.64 ± 0.06*	*k*_L_*a* 2.58 ± 0.185*
µ_max_ (h^−1^)	0.46 ± 0.02^a^	0.48 ± 0.02^a^	0.76 ± 0.22^a^
Doubling time (h)	1.52 ± 0.05^a^	1.44 ± 0.05^a^	1.04 ± 0.23^a^
Y_XS_ (g g^−1^)	0.097 ± 0.005^a^	0.100 ± 0.008^a^	0.150 ± 0.006^b^
Y_PS_ (g g^−1^)	0.256 ± 0.023^a^	0.367 ± 0.021^b^	0.256 ± 0.028^a^
Final Biomass (g L^−1^)	2.01 ± 0.02^a^	2.46 ± 0.16^a^	3.13 ± 0.17^c^
Lactic acid (g L^−1^)	5.28 ± 0.35^a^	8.71 ± 0.71^b^	5.14 ± 0.35^a^
pH	5.67 – 4.27	5.5	5.5

*Minimum *k*_L_*a* value obtained at bioreactor scale (225 rpm and 0.05 vvm). Data with the same letter at the same row are not statistically significant different, according to the Fisher’s least significant difference (LSD) procedure, at the 95.0% confidence level

### Cultivation of *L. reuteri* in a Molasses-Based Culture Medium

Figure 6B illustrates the kinetics of *L. reuteri* growing in a molasses-based medium. Growth was faster in the molasses-based culture medium than that in the MRS medium; the exponential phase for biomass growth ended at 8 h and 14 h in the economical and MRS medium, respectively. Table 2 shows the obtained kinetic parameters for both the MRS and the inexpensive medium. As observed, the biomass produced in the molasses medium was 27% larger than the biomass produced in the MRS broth.

In MRS cultivation, kinetics parameters obtained from bioreactor and flask scale experiments were similar, e.g., the specific growth rate (0.456 ± 0.016 h^−1^ and 0.481 ± 0.018 h^−1^, respectively) (Table 2). Regarding substrate consumption, there was a 75.94 ± 1.34% substrate assimilation in flask, in contrast to 86.7 ± 0.3% obtained in bioreactor experiments. Moreover, the production of lactic acid in flask and bioreactor experiments was 5.28 g L^−1^ and 8.71 g L^−1^, respectively, which is consistent with the percentage of sugar assimilation. This difference could be explained by the strict pH control (5.5) that can be achieved in the bioreactor. The pH in shake flasks varied from 5.7 to 4.3 from the beginning to the end of the culture, respectively (data not shown). Higher glucose consumption would render byproducts biosynthesis, e.g., lactic acid. High values of lactic acid and Yps in bioreactor had a statistically significant difference, according to the Fisher’s least significant difference (LSD) procedure, at a 95.0% confidence level. Notwithstanding, the final biomass from both experiments had no significant differences (*P* > 0.05) (values obtained were 2.01 ± 0.02 g L^−1^ in the flask and 2.46 ± 0.16 g L^−1^ in the bioreactor). Concerning biomass substrate yields, Yxs, values were very similar (0.097 ± 0.005 g g^−1^ and 0.100 ± 0.008 g g^−1^ for flask and bioreactor, respectively). Yields of product from the substrate (Yps) were dissimilar 0.256 ± 0.023 g g^−1^ and 0.367 ± 0.021 g g^−1^ for the flask and for the bioreactor, respectively. Once again, statistically significant differences (*P* < 0.05) might be caused by the characteristics of each culture system, shake flask, and stirred tank bioreactor, in terms of homogeneity, oxygen transfer, and pH control.

## Discussion

The strategy for bioprocess scaling up, using oxygen transfer coefficients (Ko and k_L_a), involves maintaining a constant (coefficient) value across different scales to ensure equal oxygen transfer rates [23]. As previously described, this study assessed the use of the oxygen transfer coefficient from shake flask (Ko) to scaling up (40 times) a biological process to bioreactor scale, ensuring an akin oxygen transfer coefficient k_L_a. A simple experimental setup was used to determine Ko values at small scale (100 mL) to estimate the shake flask’s oxygen transfer capacity, and in that way provide a coefficient that could describe a biological process unconstrained by factors such as geometry, agitation speed, impeller tip speed, volumetric gas flow rate, and/or power input per unit volume. Factors such as culture media composition and reactor setup during replicates were maintained as constant as possible.

The bioreactor operating conditions, i.e., agitation and aeration rates, were modified to characterize and compute the oxygen transfer coefficients (k_L_a) for scaling up purposes. Figure 4 presents three different k_L_a treatments and the subsequent changes in performance of biomass production. During the experimental setup, it was evident that the bioreactor could achieve high k_L_a values; however, obtaining values lower than 2.64 h^–1^ posed several operational challenges and thus were not assessed. At a k_L_a of 14 h^–1^, *L. reuteri* showed lower oxygen consumption followed by a sudden inhibition, thus leading to oxygen accumulation until saturation and low biomass production. At a k_L_a of 5.22 h^–1^, improved biomass production and oxygen consumption were observed. However, oxygen accumulated throughout the experiment and consumption stopped until saturation after the biomass reached the stationary phase, suggesting a cause of inhibition. Due to technical limitations, the lowest k_L_a reached was 2.64 h^–1^, which is higher than the Ko value obtained from flask experiments (2.01 h^–1^). At this k_L_a of 2.64 h^–1^, experiments showed better biomass production performance with stable oxygen consumption, no accumulation, and maintenance during the stationary phase. This suggests an adequate operational condition where the consumption rate equals the uptake rate and indicates an active metabolism without inhibition of the probiotic microorganism despite the presence of oxygen. Additionally, aerobic stress conditions have been reported as improvers of *L. reuteri* survival. *L. reuteri* DSM 17938 exhibits higher survival rates during freeze-drying stress when cultured under aerobic conditions compared to anaerobic conditions. Notably, the survival rates are significantly higher in air-sparged (61.8 ± 2.4%) and non-sparged (60.5 ± 6.4%) aerobic conditions, in contrast to nitrogen-sparged anaerobic conditions (11.5 ± 4.3%) [24]. These findings suggest that the presence of oxygen significantly enhances the survival of *L. reuteri* during the freeze-drying process. Aerobic stress conditions, therefore, appear to improve the resilience of *L. reuteri* under freeze-drying stress [24]. One observed limitation of this scaling up strategy relies on obtaining the same transfer coefficient within scales, further research on bioprocesses could state specific intervals for a safe operation of the biological system. In this sense, this study searched for operational conditions at shake flask and bioreactor scales that would allow and adequate growth of *L.reuteri* avoiding oxidative stress. At different *Ko* values (Fig. 2), biomass increased with oxygen supply; *Ko* reached a value of 2.01 h^−1^. A higher *Ko* of 2.01 h^−1^, the decrease in biomass growth suggested a toxic effect possibly caused by the presence of reactive oxygen species (ROS), e.g., H_2_O_2_. The toxicity of oxygen has been reported at high values of oxygen supply or oxygen concentration, for aerobic microorganisms [25]. *Blakeslea trispora,* cultured in shake flask at OTR values higher than 20.5 mmol h^−1^, showed high concentrations of H_2_O_2_, thus causing oxidative stress and cell damage [26]. In facultative microorganisms such as *L. paracasei*, Tian et al. tested different levels of aeration—oxygen uptake rate (OUR)—to control the production of lactic acid. They found inhibitory aeration levels at the stationary phase, where an OUR between 0.43 and 0.85 mmol L^−1^ h^−1^ (0.125–0.25 vvm) caused growth inhibition and deviation of carbon flux toward the acetoin fermentative pathway instead of lactic acid biosynthesis. The authors determined that an aeration of 0.025 vvm (OUR of 0.14 mmol L^−1^ h^−1^) allows an optimal production of lactic acid without affecting bacterial metabolism [27].

In *L. reuteri,* the response to oxidative stress is an active area of research specially in the last years, where microbiome research has gained a pivotal importance. Though differences between diverse strains and culture conditions might arise, there are essential elements involved in ROS tolerance and metabolism that include the synthesis of different isoforms of superoxide dismutase, the expression of genes involved in the synthesis of catalases and peroxidases, as well as NAP(P)H-dependent oxidoreductases and biofilm-related genes and transcriptional regulators. Nonetheless, it has been showed that *L. reuteri* does not express the enzymes superoxide dismutase and/or catalase to combat ROS [28]. A cystine-dependent pathway has been found in *L. reuteri* BR11, which participates in H_2_O_2_ and O_2_ tolerance, through a CyuC transporter and the *cyu* operon [29]. A study, carried out to demonstrate organism tolerance to H_2_O_2_ and the activation of related genes, showed that alkylhydroperoxidase (*ahpCF*), NADH oxidase (*noxE*), and methionine sulfoxide reductase (*msrB*) were expressed in *L. reuteri*. Further, DNA repair genes (*uvrABD, xthA*, *and umuC*), and genes for metal transporters (*pcl1 and pcl2*) and the peroxide-sensitive transcription factor PerR also showed different levels of expression [28].

Under anaerobic conditions, lactic acid is largely produced by *L. plantarum*; further, acetate prevails as the metabolite produced at higher concentration [30]. In the presence of oxygen, acetate is the principal metabolic product, with additional ATP synthesis and growth promotion at low rates [31, 32]. This behavior is improved with heme and menaquinone supplementation [11]. These results are quite similar to those found in the present study. For *L. reuteri*, a heterofermentative bacteria, our results suggest that lactic acid decreases in the presence of oxygen; acetate is produced at the same condition. This is a metabolic strategy used by heterofermentative LAB to gain more ATP. Acetate is produced from acetyl phosphate; it gets 2.5 ATP, and an increased biomass yield, instead of 1 ATP obtained under anaerobic condition [12, 33]. Lactic acid concentrations above 4 g L^−1^ were not found to inhibit the growth of *L. reuteri* [13]. Hence, it can be inferred that the lactic acid concentrations, found in this work, (between 4 and 9 g L^−1^), did not inhibit cell growth. In our study, a progressive reduction in lactic acid concentration and yield with oxygen transfer coefficient at the shake flask scale was found. We hypothesized that when oxygen transfer increases, *L. reuteri* shifts its metabolism from lactate to acetate so that bacteria can get more ATP.

Heterofermentative LAB can counteract the effect of oxidative stress through various mechanisms such as the accumulation of manganese, the production of superoxide dismutase or the production of enzymes as catalase, NADH peroxidase or glutathione reductase. *L. plantarum,* for instance, has the capacity to accumulate high intracellular concentrations of Mn (II) ions (above 35 mm), which acts as a superoxide eliminating system [34]. In heterofermentative LAB, oxygen acts as an alternate electron acceptor, changing the final products of metabolism [35]. For the case of *L. reuteri*, there is no information about the existence of manganese or manganese-related mechanisms. Yet, studies have showed the ability of *L. reuteri* to counteract oxidative stress, mainly against H_2_O_2_. It was found that, in the presence of oxygen and peroxide, the expression of *dha* T, involved in the synthesis of 1,3-propanediol oxidoreductase, is increased by detoxifying the system [36]. The oxygen transfer coefficient obtained in this study was similar to that found in the literature for aerobic cultures of *Lacticaseibacillus paracasei*, where coefficients between 2 h^−1^ and 4.3 h^−1^, and biomass between 7.11 g L^−1^ and 10.15 g L^−1^ were attained [37].

*L. reuteri* is aerotolerant but produces fewer antioxidant enzymes compared to other lactic acid bacteria [12]. Under experimental conditions, the oxygen transfer coefficient (k_L_a) reaches up to 2.01 ± 0.04 h^−1^ in a shake flask and 2.64 ± 0.06 h^−1^ in a stirred tank bioreactor. *L. reuteri* might have a limited antioxidant capacity. Higher values, representing larger DO availability, would result in low biomass yield.

In experiments using a molasses-based medium, an average k_L_a value of 2.58 ± 0.185 h^−1^ was obtained following the scaling-up methodology. Notably, the replicates exhibited greater variance compared to those in MRS medium, most likely due to the complex composition of the molasses-based medium, which directly influences k_L_a values [38]. Interestingly, the biomass growth was higher than that in the MRS medium. It has been considered that when glucose is replaced by sucrose as the carbon source in *L. reuteri* cultures, biomass concentration can increase as high as twice the initial value [39]. *L. reuteri* has the advantage of counting on invertase [40], and glycosyltransferase activity, which confers adaptation capacity in media without glucose [41]. Årsköld et al. [39] studied the metabolism of fructose and found that fructose cannot be used as a source of carbon. This sugar is an external electron acceptor that reduces mannitol and NADP^+^, generating more energy which might indicate an increase of biomass. Also, in a sucrose medium, expression of glycolytic enzymes is two-fold higher. Sucrose is the most abundant carbon source in sugarcane molasses. Likewise, molasses have a content of vitamins and minerals, as well as protein sources that contribute to a more enriched medium than a synthetic media such as MRS [42, 43]. Molasses also provides an environment with a greater buffer capacity, which favors diverse metabolic reactions. From this, sugarcane molasses might be a promising industrial residue to be used as carbon source in cultures of *L. reuteri*. This is namely important to reduce raw material costs in processes that include the culture of *L. reuteri* and other LAB.

The information generated in this study provides evidence of the successful use of the oxygen transfer coefficient as a scaling-up criteria. Furthermore, it demonstrates how engineering methods, such as Ko o k_L_a determinations, can characterize a biological system and facilitate scaling up with similar expected results. Interestingly, Ko or k_L_a-based strategy for scaling up has proven to be a useful tool for aerobic processes, though *L. reuteri* is an aerotolerant microorganism. Additionally, the study explored the use of sugarcane byproducts, like molasses, in biotechnological applications, promoting the circular economy. It is recommended to develop further studies focused on the search for more economical culture media for *L. reuteri*, through experimentation with different carbon and nitrogen sources from residuals of the agro-industrial sector. To the best of our knowledge, a study focused on the effect of oxygen transfer in *L. reuteri* has not been previously reported.

## Conclusion

In this study, it was found that the oxygen transfer coefficient strongly affects the growth of *L. reuteri* in shake flask and bioreactor. The use of this transfer coefficient as a scaling up parameter allowed to obtain a similar final biomass concentration in both shake and bioreactor scale (2.01 g L^−1^ and 2.46 g L^−1^). It was shown that despite being an aerotolerant bacteria, *L. reuteri* can endure diverse concentrations of oxygen and adapt its metabolism for biomass production. Considering the aerotolerant condition of *L. reuteri*, it is recommended to measure reactive oxygen species under anaerobiosis and in the different level of oxygen transfer, used in this study, as a way of explaining the limited biomass growth at high *Ko* levels in shake flasks or *k*_L_*a* in bioreactor. To the best of our knowledge, this is the first report in which the effect of oxygen transfer rate on *L. reuteri* is studied. Furthermore, the culture of *L. reuteri* in bioreactor, using an economical culture medium based on sugarcane molasses, allowed reaching a higher biomass concentration than that obtained in the MRS medium; it represents an important economic advantage for the industrial production of this probiotic. All these results are of great industrial importance, since the microbial biomass obtained from LAB is used as a probiotic matrix for many human and veterinary products. *L. reuteri* is a probiotic bacterium that synthesizes a metabolite of high antimicrobial power, reuterin, under anaerobic conditions. The information generated in this study will provide the basis for the design of a process whose purpose would be the production of probiotics or reuterin, considering its oxygen requirements.
